# Extraskeletal Ewing's Sarcoma Arising from the Sciatic Nerve: A Diagnostic Challenge

**DOI:** 10.1155/2015/172635

**Published:** 2015-07-08

**Authors:** Aadhar Sharma, Kate Brown, John Skinner, Jeremy Whelan, Michael Fox

**Affiliations:** ^1^Peripheral Nerve Injury Unit, Royal National Orthopaedic Hospital, Stanmore, Middlesex HA7 4LP, UK; ^2^Sarcoma Unit, Royal National Orthopaedic Hospital, Stanmore, Middlesex HA7 4LP, UK; ^3^Department of Clinical Oncology, University College Hospital, Euston Road, London NW1 2BU, UK

## Abstract

Ewing's sarcoma is a common bone tumour of childhood but is a rare occurrence in individuals over 20 years of age. Few cases are reported as originating from peripheral nerves. We present an unusual case of extraosseous Ewing's sarcoma originating from the sciatic nerve in a 66-year-old patient which had the clinical hallmarks of a benign nerve sheath tumour. Following discussion at a multidisciplinary meeting, excision biopsy of the suspected benign nerve sheath tumour was planned. At operation, the mass had malignant features. Histology confirmed the presence of Ewing's sarcoma. Due to the morbidity of nerve resection, radiotherapy and chemotherapy were commenced. Ewing's sarcoma is known to mimic benign pathologies. In this case there were subtle signs of a malignant process in the form of unremitting pain. It is vital to keep in mind the less common tumours that can affect the peripheral nervous system in such cases.

## 1. Introduction

Ewing's sarcoma is a malignant round cell tumour most commonly originating in bone in adolescents and young adults. It is predominantly found in patients below the age of 20 years and has a slight male predilection [1]. The Ewing sarcoma family of tumours, including primitive neuroectodermal tumours and Askin tumours as well as Ewing's sarcoma, represent the second most common primary bone tumour of childhood [2]. Its extraosseous variant is very rare with few cases reported as originating from peripheral nerves [3–8]. Benign tumours arising from peripheral nerves may present clinically with motor and/or sensory dysfunction and a positive Tinel's sign. Unremitting pain refractory to analgesia is however more suggestive of malignancy [9].

We present an unusual case of extraosseous Ewing's sarcoma (EES) arising from the sciatic nerve of a patient referred to our tertiary peripheral nerve injury unit and discuss the issues surrounding its management.

## 2. Case Report

**Figure 1:**
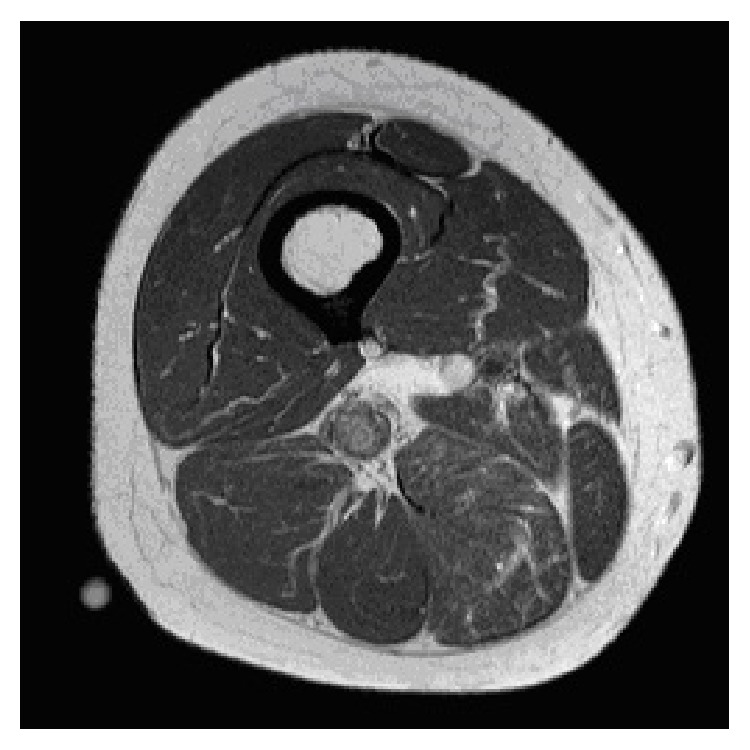
Axial MRI imaging of the right thigh demonstrating the mass originating from the sciatic nerve.

A 66-year-old teacher with no past medical history of note presented with a 9-month history of worsening paraesthesia on the lateral aspect of her right foot and pain in her right thigh. She had Tinel's sign on percussion of the sciatic nerve in the mid-thigh with radiation into the foot. Magnetic resonance imaging (MRI) revealed a 1.4 × 1.4 × 3 cm soft tissue mass originating from the right sciatic nerve 15 cm proximal to the knee (Figure 1). Following established protocol, the multidisciplinary team consisting of musculoskeletal radiologists and orthopaedic sarcoma specialists reviewed the clinical picture and the MRI images. The likelihood of malignancy was deemed to be low with a provisional diagnosis of a schwannoma and the decision was made to proceed with excision biopsy.

**Figure 2:**
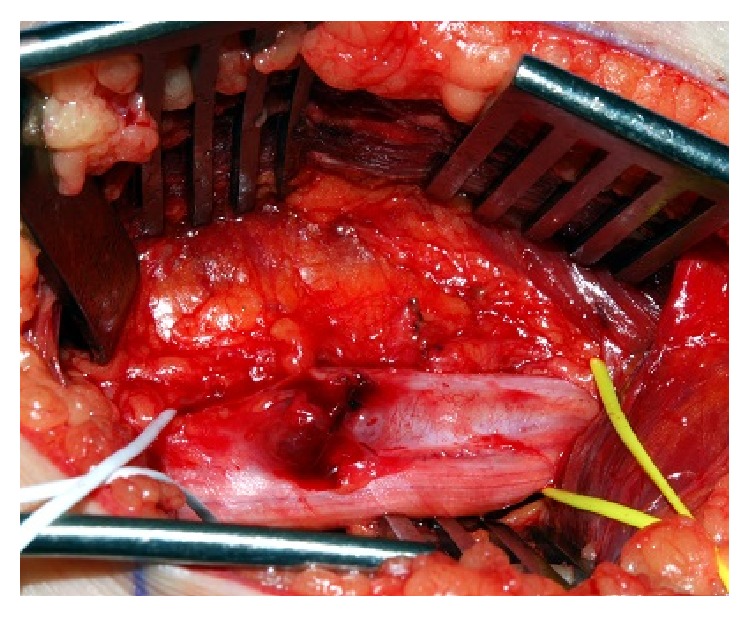
Intraoperative photo of Ewing's sarcoma arising from the tibial segment of the right sciatic nerve (controlled with slings) in the mid-thigh. The areas of necrosis as well as the irregular shape of the lesion clearly differentiate it from the appearances of a schwannoma.

Intraoperative appearances were of a hard tumour with poorly defined borders that was infiltrative into the fascicles of the tibial component of the sciatic nerve. These are appearances more consistent with those of a malignant lesion (Figure 2). Therefore the opinion of a specialist sarcoma surgeon was sought intraoperatively. Rather than to proceed with excision, a wedge biopsy was performed and samples were sent for histological analysis.

**Figure 3:**
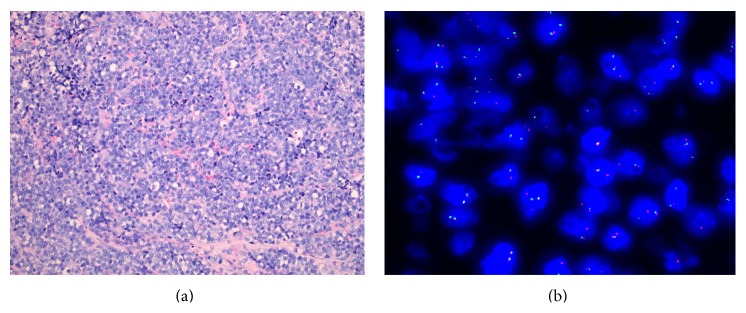
(a) Microphotograph of intraneural Ewing's sarcoma showing sheets of monotonous small round cells (10x magnification; H&E stain). (b) FISH study of the intraneural Ewing's sarcoma. The split red dots indicate a rearrangement of the EWS gene.

Histology showed changes consistent with Ewing's sarcoma with pathognomonic sheets of round cells having a high nuclear to cytoplasmic ratio (Figure 3(a)). While immunochemistry showed strong positivity for CD99, this is not considered specific for Ewing's sarcoma. Diagnosis was confirmed upon fluorescent in situ hybridisation (FISH) testing which revealed a rearrangement of the EWS gene (Figure 3(b)), most commonly occurring due to translocation of EWSR1, a fusion transcription factor found in 85% of Ewing's family tumours [10].

Further investigation revealed no evidence of metastatic disease. Surgical management of this lesion classically involves neurectomy of the sciatic nerve and a wide excision of surrounding tissues [1]. Radical excision would involve resection of the whole affected limb compartment [1]. Both these treatments would result in significant morbidity and therefore the decision has been made for the patient to undergo radiotherapy and chemotherapy with the hope of eradicating the tumour and preserving limb function with the knowledge that the evidence also shows that Ewing's sarcoma is sensitive to these treatment modalities [2].

## 3. Discussion

Ewing first described a long bone tumour consisting of undifferentiated round cells in 1921; a tumour with similar histological appearances originating from a peripheral nerve had already been described in 1918; however it was not until 1975 that the concept of an EES began to take shape [11–13]. The population in which it is most commonly found is adolescents and young adults with fewer than 20% of diagnoses being made in patients aged over 20 years [1]. Leiomyosarcoma, liposarcoma, and undifferentiated sarcoma are the variations more commonly found in the older adult population [14]. Since intraneural Ewing's sarcoma was first described it has only sporadically been reported ever since. This case therefore represented a diagnostic dilemma in that the tumour in question was located in the sciatic nerve of a patient outside of the age range characteristically associated with Ewing's sarcoma.

This case report underlines the importance of correlating clinical findings with available imaging to formulate differential diagnoses [15]. Diagnosis of Ewing's sarcoma has been known to present a challenge owing to its ability to mimic benign pathological processes. In this case however, the history included unremitting pain in the right thigh that was refractory to analgesia as well as neuropathic pain in the foot. This raises the suspicion of both a pathological process involving the tibial nerve and a malignant lesion [9].

Treatment of Ewing's sarcoma in most cases involves a multimodal approach involving neoadjuvant and adjuvant chemotherapy, radiotherapy, and wide local excision. Given a lack of high level evidence supporting particular modalities for treating a primary lesion however, the choice of management remains with the treating clinician [1]. In this case, wide local excision would involve neurectomy of the sciatic nerve which would result in significant morbidity for the patient and hence the choice of nonsurgical management. The vital role of chemotherapy in the management of Ewing's sarcoma cannot be overemphasised. Prior to the use of chemotherapy in its management, survival rates stood at approximately 10%, since its widespread introduction survival has drastically improved to 75%, and its impact and subsequent importance are therefore clear [2].

Ewing's sarcoma is well documented to be an aggressive high grade tumour and therefore requires management to be expedited under the care of a regional specialist sarcoma unit [1, 3]. Studies have shown that prognosis is particularly poor if metastases are present at the point of diagnosis [1, 2]. The ability of EES to mimic benign peripheral nerve sheath tumours is well acknowledged [10].

The clinical picture of EES has been reported as a rapidly growing, painful tumour affecting most commonly the lower limb and paravertebral region [15]. While the role of radiography has been more to exclude rather than to diagnose EES, our case report underlines the importance of using not only radiology but also the clinical presentation in making a diagnosis whilst remembering to keep in mind the less common tumours that can affect the peripheral nervous system [16]. In this case, the unremitting nature of the neuropathic pain was suggestive of a rapidly growing, infiltrative lesion.
